# The association pathway between music practice and mental health: a study on social connectedness, perceived stress, and anxiety among individuals with piano experience

**DOI:** 10.3389/fpsyg.2026.1957899

**Published:** 2026-09-08

**Authors:** Jie Tan

**Affiliations:** Music College of Sichuan Normal University, Chengdu, Sichuan, China

**Keywords:** anxiety, cross-sectional study, flow experience, music practice, perceived stress, social connectedness.

## Abstract

**Objective:**

To explore the association pathway between daily participation in music practice and mental health indicators, focusing on analyzing the cross-sectional network relationships among performing flow experience, social connectedness, perceived stress, and anxiety among individuals with piano experience.

**Methods:**

A cross-sectional online survey design was adopted, yielding a total of 890 valid questionnaires. Based on the presence or absence of piano practice experience, the sample was divided into two questionnaire coding groups: Group *A* (no experience/stopped practicing, *n* = 318) and Group *B* (has experience/currently practicing, *n* = 572). Independent sample t-tests were used to compare differences in mental health indicators between the groups. Within Group *B*, Pearson correlation analysis and Bootstrap resampling techniques were employed to test specific serial indirect effects and the moderating role of practice years.

**Results:**

(1) The social connectedness of Group *B* was significantly higher than that of Group *A*, and their perceived stress and anxiety were significantly lower than those of Group *A*, but the effect sizes of the group differences were small; (2) Within Group *B*, flow experience was significantly positively correlated with social connectedness, while social connectedness was significantly negatively correlated with stress and anxiety, and flow had no significant direct correlation with stress and anxiety; (3) The serial indirect effect model supported the significance of the specific indirect pathway of “flow experience → social connectedness → perceived stress → anxiety” (the confidence interval did not contain 0), but the total indirect effect and the moderating effect of practice years did not receive significant statistical support.

**Conclusion:**

The relationship between flow experience and lower stress or anxiety in piano practice is not a simple direct mapping, but constitute a specific cross-sectional statistical association pathway through the potential joint transformation of social connectedness and perceived stress. The results provide empirical clues for exploring the associations between daily arts participation and mental health.

## 1 Introduction

Individuals in modern society face varying degrees of psychological stress, and perceived stress is typically associated with anxiety. Continuous tension and psychological load may be linked to an individual’s daily functioning and the need for mental health services. Therefore, in addition to professional mental health services, activities that can be integrated into daily life have gradually attracted the attention of psychology and public health research (Mak et al., 2021).

Forms of arts participation are relatively flexible and can be carried out in daily and non-clinical contexts. Existing research has discussed the relationship between arts activities and emotional experience, social participation, and subjective wellbeing, and music activities have also been used to examine emotions, physiological arousal, and stress responses (Fancourt et al., 2020; MacDonald, 2014). Even in different stages of life and specific contexts (such as behavioral development in youth or interventions during specific physiological periods), music participation has shown effects on multidimensional psychological states (Bogt et al., 2021; Wulff et al., 2021); recent large-scale meta-analyses have also empirically demonstrated the significant improving role of music interventions on health-related quality of life, and active participation forms are often accompanied by specific benefits (McCrary et al., 2022). However, existing music psychology research focuses more on music listening in family and daily scenarios (Krause and North, 2016; Shan et al., 2024), and there is still room for research on the psychological changes of practitioners and enthusiasts resulting from active instrumental practice (such as orchestra participation or personal instrument practice) (Derbyshire et al., 2021). Furthermore, the links between musical activity and psychosocial wellbeing are multifaceted. Physiologically, active music-making has been associated with the modulation of neurohormones such as oxytocin, which plays a critical role in facilitating social bonding and connectedness (Gebauer et al., 2016). Psychologically, concepts such as alexithymia—the inability to identify and communicate emotions—are crucial in understanding social connectedness and have been hypothesized to mediate the relationship between music training and social functioning. Additionally, genetic predispositions are fundamental factors that influence both music practice persistence and related psychological traits (Ullén et al., 2016).

Piano practice involves score recognition, auditory feedback, motor control, and sustained attention (Altenmüller and Schlaug, 2015), and may occur in contexts such as personal practice, teacher-student instruction, collaborative performance, and public performance. These activity characteristics may be associated with flow experience, social connectedness, perceived stress, and anxiety. However, piano experience may also be simultaneously correlated with family resources, educational background, personal interests, time conditions, and pre-existing psychological states, and cross-sectional group differences cannot be interpreted as the effect of piano practice.

Based on this, this study uses cross-sectional online questionnaire data to examine whether there are differences in social connectedness, perceived stress, and anxiety among piano experience coding groups, and analyzes the correlations, specific serial indirect associations, and the moderating effect of practice years among flow experience, social connectedness, perceived stress, and anxiety within Group *B*.

## 2 Theoretical framework and research hypotheses

### 2.1 Music practice and mental health

Music practice is not only an aesthetic activity but also an active participation process that requires sustained attention, motor coordination, and emotional involvement. Compared with passive music listening, instrumental practice usually involves repeated operations, immediate auditory feedback, and skill adjustment, and may be associated with relatively stable task engagement and self-regulation experiences (Dingle et al., 2021; Thoma et al., 2013). Existing studies have shown that arts participation may be positively correlated with emotional regulation, social participation, and subjective wellbeing (Agres and Chen, 2025); music activities may also be linked to individuals’ stress responses and social connection experiences.

Piano practice usually possesses attributes of both personal practice and social participation. On the one hand, individuals can obtain focused experiences in relatively independent practice contexts; on the other hand, piano learning may also involve teacher guidance, peer communication, family sharing, public performance, and music community participation. These activity characteristics may be correlated with an individual’s social connectedness, perceived stress, and anxiety. Therefore, this study first examines whether there are differences in the aforementioned psychological variables among different piano experience groups, and proposes the following hypotheses:

*H1a*: The social connectedness of Group *B* is higher than that of Group *A*.

*H1b*: The perceived stress of Group *B* is lower than that of Group *A*.

*H1c*: The anxiety of Group *B* is lower than that of Group *A*.

### 2.2 Flow experience

Flow experience refers to the highly focused state, merging of action and awareness, altered time perception, and intrinsic reward experience that may occur when individuals engage in activities where skill requirements match their capabilities (Csikszentmihalyi, 1990). Piano practice requires performers to synchronously process multiple musical information, providing an ideal context for the formation of flow. The self-transcendence experience generated during this process is believed to be closely related to an individual’s wisdom, intrinsic satisfaction, and wellbeing (Kim et al., 2023). Because piano practice usually involves continuous daily participation, the positive state experience individuals gain in typical performances may also be linked to their more stable general psychological performance. Research also indicates that in music and sports activities, higher flow experience helps individuals maintain good psychological adaptation when coping with life stress (Habe et al., 2021).

Theoretically, higher task engagement may manifest as individuals focusing more attention on the current activity and may be linked to their immediate emotional experiences. Therefore, this study uses flow experience as the starting variable of the theoretical model to examine its relationship with social connectedness, perceived stress, and anxiety. Flow experience has strong intrinsic motivation attributes. Individuals who report a stronger sense of skill matching and intrinsic satisfaction during performance may also show higher intentions for continuous participation and be more inclined to share performance experiences or participate in related social interactions (Savage et al., 2020). Therefore, a stronger flow experience may be associated with higher social connectedness. At the same time, characteristics such as concentration of attention, clear goals, and immediate feedback contained in the flow state may be related to an individual’s degree of attention to stress cues and negative emotions. Thus, the following hypotheses are proposed:

*H2a*: In Group *B*, flow experience is positively correlated with social connectedness.

*H2b*: In Group *B*, flow experience is negatively correlated with perceived stress.

*H2c*: In Group *B*, flow experience is negatively correlated with anxiety.

### 2.3 Social connectedness, perceived stress, and anxiety

Social connectedness is the psychological experience of an individual subjectively feeling understood, accepted by others, and belonging to a certain social network. Existing research generally believes that higher social connectedness is related to better psychological adaptation and lower loneliness, and may be correlated with an individual’s stress appraisal and emotional regulation states (Perkins et al., 2021). Although piano practice is often conducted in the form of individual activities, it may also involve teacher-student interactions, peer communication, family sharing, public performances, and online music communities. These social contexts may associate piano practice with an individual’s general social connectedness. The stress-buffering perspective of social support suggests that the stable social connections and understanding, recognition, and support perceived by individuals may be related to their ways of appraising stressful situations (Greenberg et al., 2021; Tarr et al., 2014); higher social connectedness may also be accompanied by lower feelings of uncontrollability and unpredictability. Therefore, higher social connectedness may be correlated with lower perceived stress. Perceived stress reflects an individual’s subjective appraisal of recent life load, uncontrollability, and lack of coping resources. An individual’s appraisal of the uncontrollability and difficulty of coping with daily events is usually correlated with anxiety symptoms such as tension, worry, difficulty relaxing, and irritability. Accordingly, the following hypotheses are proposed:

*H2d*: In Group *B*, social connectedness is negatively correlated with perceived stress.

*H2e*: In Group *B*, social connectedness is negatively correlated with anxiety.

*H2f*: In Group *B*, perceived stress is positively correlated with anxiety.

### 2.4 Specific serial association pathway

Synthesizing flow theory, the social connectedness perspective, and stress-buffering theory, this study proposes a specific serial association pathway: “flow experience—social connectedness—perceived stress—anxiety.”

This theoretical model contains three sets of interconnected variable relationships. First, according to the broaden-and-build theory of positive emotions (Fredrickson, 2001) and the psychological spillover effect, the state-like flow experience cultivated during specific piano practices can help individuals accumulate and expand their psychological and social resources over time. Therefore, stronger flow experiences in daily practice may spill over into more positive general music participation. This positive state is often accompanied by higher intentions for continuous participation, expression, and sharing, which in turn show an association with higher trait-like social connectedness. Second, higher social connectedness reflects an individual’s stronger experience of belonging, understanding, and support, and may be correlated with lower perceived stress. Third, lower perceived stress may be correlated with less anxiety.

Accordingly, flow experience, social connectedness, perceived stress, and anxiety may present a specific serial indirect association. The variable order in this sequence is set based on theoretical relationships, and their statistical association is tested through cross-sectional data. Thus, the following hypothesis is proposed:

*H3*: In Group *B*, flow experience, social connectedness, perceived stress, and anxiety present a specific serial indirect association, namely, the specific serial indirect effect of “flow experience → social connectedness → perceived stress → anxiety” is not 0.

### 2.5 The moderating role of practice years

Individuals with longer practice years may have higher performance familiarity and relatively more stable ways of music participation. Richer practice experience may also be accompanied by more teacher-student interactions, peer communication, performance sharing, and music community participation, and correlated with a closer connection between personal performance experience and social interactions.

Compared with individuals with shorter practice years, those with longer practice years may have more stable performance skills and richer music participation experience. Therefore, among individuals with longer practice years, the connection between flow experience and music identity and social interaction experience may be tighter, and the positive association between flow experience and social connectedness may also be stronger. Accordingly, the following hypothesis is proposed:

*H4*: Practice years moderate the relationship between flow experience and social connectedness, manifesting as the longer the practice years, the stronger the positive association between the two.

**FIGURE 1:**
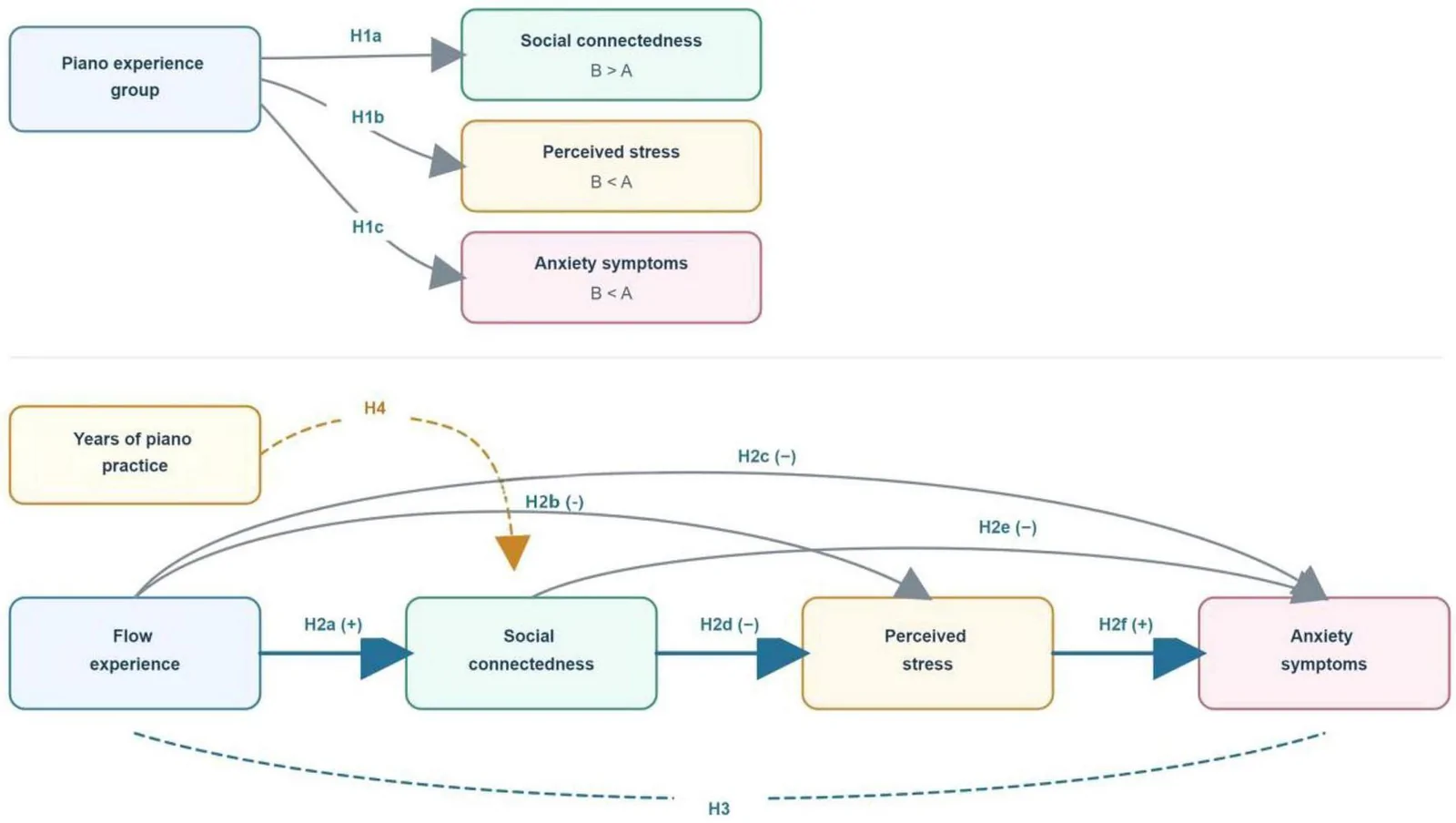
Theoretical framework of the relationship between music practice and mental health. Solid lines in the figure represent main hypothesized paths in the theoretical model, gray curved lines represent supplementary hypothesized paths, the orange dashed line represents the moderating hypothesis of practice years, and the blue dashed line represents the specific serial association hypothesis. Arrows represent the statistical association direction under theoretical settings, not denoting time sequence or causal relationships. H1a–H1c represent hypotheses on piano experience group differences, H2a–H2f represent hypothesis on core variable associations in Group *B*, H3 represents the specific serial indirect association hypothesis, and H4 represents the practice years moderation hypothesis.

Based on the above theoretical analysis, this study constructed a theoretical framework with music practice experience as the background, flow experience as the starting variable, social connectedness and perceived stress as sequential mediating variables, and anxiety as the outcome variable. The study simultaneously examines the psychological variable differences of piano experience groups, the associations between core variables within Group *B*, the specific serial association formed by social connectedness and perceived stress, and the moderating effect of practice years on the relationship between flow experience and social connectedness. The specific theoretical model and research hypothesis paths are shown in Figure 1.

## 3 Research methods

### 3.1 Research design and sample

**FIGURE 2:**
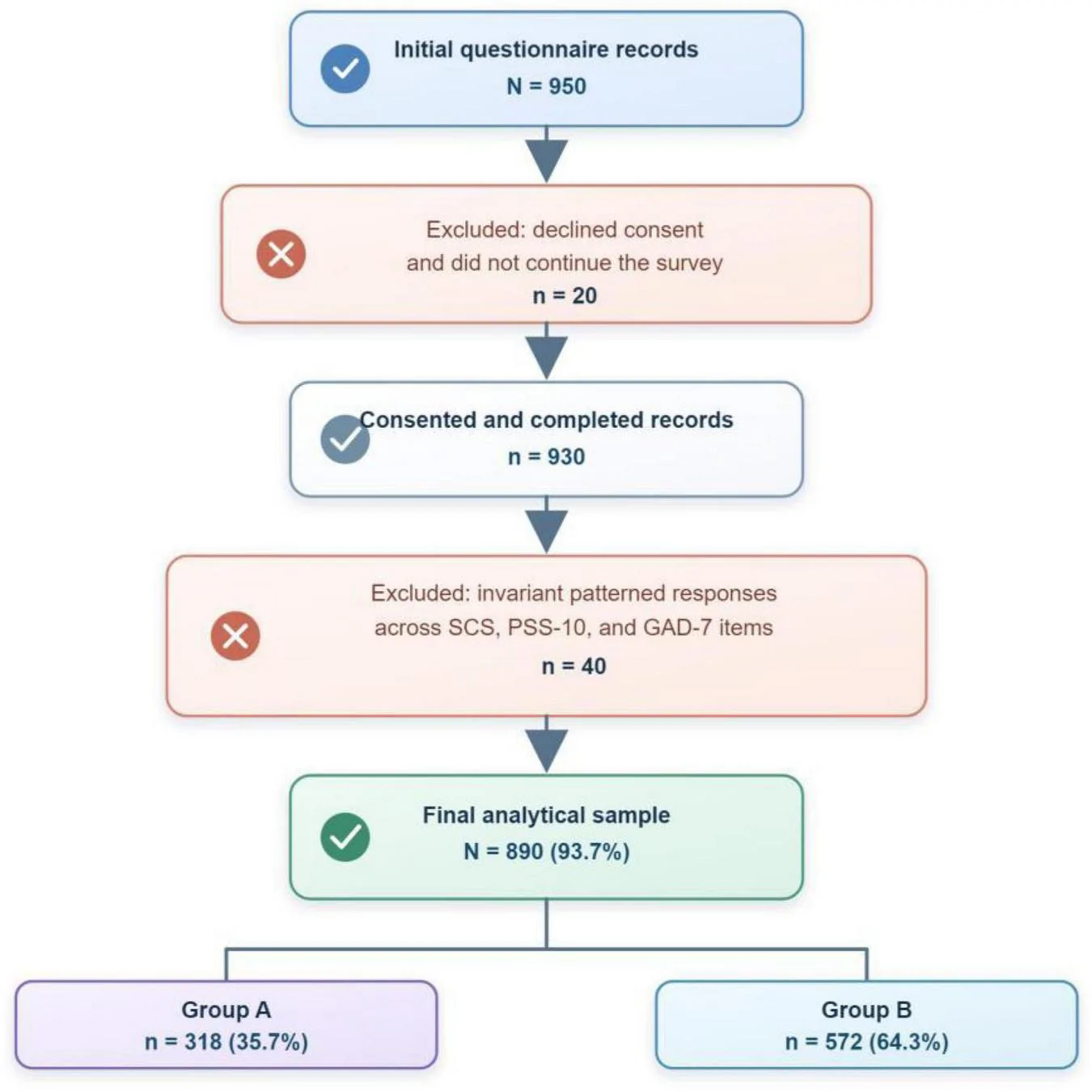
Research sample screening flowchart. Unengaged responses refer to providing identical ratings (3) for all items in the brief Social Connectedness Scale, identical ratings (2) across the PSS-10, and identical ratings (1) across the GAD-7 in the original responses.

This study adopted a cross-sectional online questionnaire survey design. Data were collected online via the Wenjuanxing platform (a professional online survey platform in China) from March to June 2026. The questionnaire links were published through social media and online communities, recruiting respondents using convenience sampling, yielding a total of 950 questionnaire records. The first page of the questionnaire explained the research purpose, the principle of voluntary participation, and anonymous confidentiality methods, and respondents could only continue answering after confirming informed consent. During data cleaning, 20 records that did not consent to participate and did not continue answering were excluded first; subsequently, based on the data quality standards set in this study, 40 records with unengaged responses—such as providing identical ratings (3) for all items in the brief Social Connectedness Scale, identical ratings (2) across the 10-item Perceived Stress Scale (PSS-10), and identical ratings (1) across the Generalized Anxiety Disorder-7 (GAD-7)—were excluded. Ultimately, 890 valid questionnaires were included, with a sample inclusion rate of 93.7%. The sample screening process is shown in Figure 2.

In the final sample, there were 318 people in Group *A*, accounting for 35.7%; and 572 people in Group *B*, accounting for 64.3%. The respondents had a mean age of 24.6 years (*SD* = 6.2, ranging from 18 to 45 years). The piano experience groups were divided according to the actual choices made by respondents in the questionnaire: those who chose “never learned / completely do not play currently” were classified as Group *A*, and those who chose “have piano experience / currently practicing” were classified as Group *B*. Since the questionnaire options simultaneously involve past experience and current practice status, this paper uses the neutral “Group *A*” and “Group *B*” for expression, and interprets between-group results as differences between questionnaire coding groups. The basic characteristics and piano experience group distribution of the final sample are shown in Table 1.

**TABLE 1:** Basic characteristics and piano experience group distribution of respondents (*N* = 890).

Demographic variable	Category	Frequency (n)	Percentage (%)
Age (years)	Mean ± *SD*	24.6 ± 6.2	–
Gender	Male	373	41.9
	Female	517	58.1
Education level	High school/technical school and below	93	10.4
	Associate degree	176	19.8
	Bachelor’s degree (including current students)	435	48.9
	Master’s degree and above (including current students)	186	20.9
Current occupational status	Full-time student	144	16.2
	Full-time job	526	59.1
	Freelancer/part-time	141	15.8
	Unemployed/retired	79	8.9
Piano experience	Group *A*	318	35.7
	Group *B*	572	64.3

Group *A* comprises respondents who chose “never learned/completely do not play currently”; Group *B* comprises respondents who chose “have piano experience/currently practicing”. Percentages may not add up exactly to 100.0% due to rounding.

Piano practice years, recent practice frequency, and flow experience items were only filled out by respondents in Group *B*. Therefore, the null values for these variables in Group *A* belong to structural missingness produced by the questionnaire’s skip logic design. Analyses involving piano practice characteristics and flow experience were all conducted within Group *B*. The distribution of piano practice years and recent practice frequency of Group B respondents are shown in Table 2.

**TABLE 2:** Piano practice characteristics in group B (*N* = 572).

Characteristic variable	Category	Frequency (*n*)	Percentage (%)
Practice years	Within 1 year	177	30.9
	1–3 years	189	33.0
	3–5 years	95	16.6
	Over 5 years	111	19.4
Recent practice frequency	Less than once a week	119	20.8
	1–2 times a week	224	39.2
	3–4 times a week	136	23.8
	5 times or more a week	93	16.3

Practice years and recent practice frequency were only filled out by Group *B* respondents. Percentages may not add up exactly to 100.0% due to rounding.

### 3.2 Measurement tools

The measurement contents of this study include piano practice characteristics, flow experience, social connectedness, perceived stress, and anxiety. Piano practice characteristics are described by practice years and recent practice frequency; flow experience, social connectedness, perceived stress, and anxiety are assessed using corresponding measurement tools. The brief Flow Experience Scale uses 5-point scoring, the brief Social Connectedness Scale uses 6-point scoring, PSS-10 uses 5-point scoring, and GAD-7 uses 4-point scoring. The scores of items in each scale are added to form a total score. Specific scoring methods are detailed below, and full items are found in Supplementary material.

For the flow and social connectedness scales, which were abbreviated for this study, items were primarily selected based on their core theoretical relevance and high factor loadings from their original established tools. A standard translation and back-translation procedure was strictly followed. Furthermore, a small-scale pilot test involving a preliminary group of Chinese piano practitioners was conducted prior to the formal survey to ensure item clarity and cultural appropriateness. While these specific short-forms lack extensive prior validation, their structural validity is robustly corroborated by the confirmatory factor analysis (CFA) results in the present study.

#### 3.2.1 Piano practice characteristics and flow experience

Piano practice characteristics include practice years and recent practice frequency. Practice years are divided into four categories: “within 1 year,” “1–3 years,” “3–5 years,” and “over 5 years”; recent practice frequency is divided into four categories: “less than once a week,” “1–2 times a week,” “3–4 times a week,” and “5 times or more a week.” These indicators respectively reflect the respondents’ piano practice experience and recent participation frequency.

The measurement of flow experience is based on Csikszentmihalyi’s (1990) flow theory, referring to research on flow measurement tools by Jackson et al. (2008), and compiled in combination with the piano performance context. The brief Piano Performance Flow Experience Scale contains 5 items, covering challenge-skill balance, merging of action and awareness, concentration on the task at hand, transformation of time, and autotelic experience.

The scale instructions ask respondents to recall a typical fully engaged playing state and answer according to their subjective experience in that context. Each item is scored from 1 to 5 points. The scores of the 5 items are added up to form a total flow experience score, ranging from 5 to 25 points. Higher scores indicate stronger piano performance flow experience reported by the respondent.

#### 3.2.2 Brief social connectedness scale

The measurement of social connectedness refers to the social connectedness theory and related measurement tools proposed by Lee and Robbins (1995), adjusted in combination with the questionnaire length and survey context of this study. Social connectedness reflects the degree of belonging, understanding, acceptance, and interpersonal connection individuals feel in general social relationships.

The brief Social Connectedness Scale used in this study contains 7 items, scored 1–6. Items 1, 3, 5, and 7 are reverse-scored using the formula 7–*x*. The scores of the 7 items are added to form a total social connectedness score, ranging from 7 to 42 points. Higher scores indicate stronger social connectedness reported by the respondent.

#### 3.2.3 Perceived stress scale

Perceived stress was measured using the 10-item Perceived Stress Scale (PSS-10) developed by Cohen et al. (1983). This scale is used to assess individuals’ subjective feelings about the unpredictability, uncontrollability, and psychological load of life events over the past month.

PSS-10 contains 10 items, scored 0-4. Items 4, 5, 7, and 8 are reverse-scored using the formula 4–x. The scores of the 10 items are added to form a total perceived stress score, ranging from 0 to 40 points. Higher scores indicate higher perceived stress reported by the respondent.

#### 3.2.4 Generalized Anxiety Disorder Scale

Anxiety was measured using the Generalized Anxiety Disorder-7 (GAD-7) developed by Spitzer et al. (2006). This scale contains 7 items and is used to evaluate the frequency of respondents experiencing symptoms such as feeling nervous, not being able to stop worrying, worrying too much, trouble relaxing, being so restless that it is hard to sit still, becoming easily annoyed or irritable, and feeling afraid as if something awful might happen, over the past 2 weeks.

Each item is scored 0–3, representing “Not at all,” “Several days,” “More than half the days,” and “Nearly every day,” respectively. The scores of the 7 items are added to form a total anxiety score, ranging from 0 to 21 points. Higher scores indicate more obvious anxiety reported by the respondent. This study included the GAD-7 total score as a continuous variable in statistical analysis.

### 3.3 Statistical analysis

Data review and statistical analyses were completed using Python 3.11, primarily using pandas, SciPy, statsmodels, and semopy. First, frequencies, percentages, means, and standard deviations of the variables were calculated, and Cronbach’s α was used to evaluate the internal consistency of each scale. Structural missingness in Group A caused by skip logic was not imputed.

Because flow experience items were only filled out by Group *B* respondents, preliminary tests for common method bias and measurement model testing were conducted among the 572 respondents in Group *B*. Harman’s single-factor test implemented an unrotated principal component analysis based on 29 measurement items. Subsequently, a four-factor confirmatory factor analysis was conducted on flow experience, social connectedness, perceived stress, and anxiety. Model parameters were estimated using the Maximum Likelihood (ML) method, and fit indices such as χ^2^, χ^2^/*df*, CFI, TLI, RMSEA, GFI, AGFI, and NFI were reported. Composite Reliability (CR) and Average Variance Extracted (AVE) were calculated based on standardized factor loadings, and discriminant validity was evaluated using the Fornell-Larcker criterion.

H1a-H1c used independent sample *t*-tests to compare differences in social connectedness, perceived stress, and anxiety between Group A and Group *B*. Before testing, Levene’s test was used to examine the homogeneity of variance, and Cohen’s *d* as well as the 95% confidence intervals of the between-group mean differences were reported. Mean differences were calculated as Group A minus Group *B*.

H2a-H2f used Pearson correlation analysis to test the bivariate relationships among flow experience, social connectedness, perceived stress, and anxiety in Group *B*. For the 6 correlation tests among the four variables, the Holm method was used to correct the *p*-values for multiple comparisons. As a supplementary analysis, considering that both practice years and recent practice frequency are ordinal categorical variables, Spearman rank correlation analysis was adopted to examine the relationships between the two piano practice indicators and flow experience, social connectedness, perceived stress, and anxiety.

H3 was tested using a serial indirect effect model, with flow experience as the independent variable, social connectedness and perceived stress as sequentially ordered mediating variables, and anxiety as the outcome variable, controlling for gender, education level, and occupational status. Categorical covariates were dummy coded. The 95% Percentile Confidence Intervals (Percentile CI) for direct effects, specific indirect effects, total indirect effects, and total effects were estimated using 5,000 case Bootstrap resampling; when the confidence interval did not contain 0, the corresponding effect was considered to have reached statistical significance. Given that this study adopted a cross-sectional survey design, the serial indirect effect was only used to test the statistical indirect association when variables were arranged according to theoretical order, and not as evidence of time sequence or causal association.

H4 was tested using interactive effects in multiple linear regression, with social connectedness as the dependent variable, controlling for gender, education level, and occupational status. First, practice years were encoded as an ordinal variable from 1 to 4, mean centering was performed on flow experience and practice years, and their interaction term was constructed. The moderating effect was judged based on the regression coefficient of the interaction term, the 95% confidence interval, and Δ*R*^2^, where Δ*R*^2^ represents the increment in explanation rate generated by adding the interaction term based on the model containing covariates and main effects. Subsequently, practice years were used as a categorical variable, with “within 1 year” as the reference group, and interaction terms were constructed through corresponding dummy variables and the centered flow experience, and joint tests were conducted on multiple interaction terms.

All statistical tests were two-tailed, and the significance level was set at *p* < .05. Unless otherwise specified, regression analyses report unstandardized regression coefficients *B*, standard errors, 95% confidence intervals, and *p*-values.

## 4 Results

### 4.1 Common method bias and internal consistency

Since flow experience items were only completed by respondents in Group *B*, the preliminary test for common method bias was conducted on the 29 measurement items from the 572 respondents in Group *B*. The results of Harman’s single-factor test are shown in Table 3. The unrotated principal component analysis extracted 4 components with eigenvalues greater than 1. The first component explained 22.08% of the total variance, which is below the 40% threshold, and there was no single component explaining the majority of the variance. This result indicates that no obvious single method factor was found in the data, but Harman’s single-factor test can only serve as a preliminary judgment for common method bias.

**TABLE 3:** Harman’s single-factor test results (Group *B*, *N* = 572).

Component	Eigenvalue	Variance explained (%)	Cumulative explanation (%)
1	6.403	22.08	22.08
2	3.162	10.90	32.98
3	2.435	8.40	41.38
4	1.926	6.64	48.02

Internal consistency test results showed that in the full sample, the Cronbach’s α for the brief Social Connectedness Scale, PSS-10, and GAD-7 were 0.824, 0.854, and 0.831, respectively. In Group *B*, the Cronbach’s α for the brief Flow Experience Scale, brief Social Connectedness Scale, PSS-10, and GAD-7 were 0.782, 0.825, 0.849, and 0.833, respectively, all reaching acceptable levels.

### 4.2 Confirmatory factor analysis

A four-factor confirmatory factor analysis was conducted among the 572 respondents in Group *B*. Model fit results were: χ^2^ (371) = 397.735, *p* = 0.163, χ^2^/*df* = 1.072, CFI = 0.994, TLI = 0.994, RMSEA = 0.011, GFI = 0.920, AGFI = 0.913, NFI = 0.920. The fit indices indicated that the four-factor measurement model fitted well with the data.

The standardized factor loadings, composite reliability, and average variance extracted for the four constructs are shown in Table 4. The Composite Reliability (CR) for each construct was above 0.78, indicating good composite reliability; AVE ranged between 0.360 and 0.418. According to classical measurement standards, when the composite reliability of a construct is higher than 0.60, even if AVE is lower than 0.50, its convergent validity is still within an acceptable range (Fornell and Larcker, 1981).

**TABLE 4:** Reliability and validity of the four-factor measurement model (Group *B*, *N* = 572).

Construct	Items	Standardized loading range	Cronbach’s α	CR	AVE
Flow experience	5	0.604–0.685	0.782	0.782	0.418
Social connectedness	7	0.606–0.669	0.825	0.825	0.403
Perceived stress	10	0.571–0.634	0.849	0.849	0.360
Anxiety	7	0.620–0.662	0.833	0.833	0.416

CR stands for Composite Reliability, AVE stands for Average Variance Extracted.

### 4.3 Descriptive statistics, discriminant validity, and correlation analysis

The means, standard deviations, Pearson correlation coefficients, and discriminant validity test results of the main variables in Group B are shown in Table 5. As shown on the diagonal of Table 5, the square roots of AVE for each construct ranged between 0.600 and 0.647, all higher than the absolute values of the Pearson correlation coefficients between the respective constructs, indicating good discriminant validity among these four constructs. Secondly, regarding the correlations between variables, flow experience was significantly positively correlated with social connectedness; social connectedness was significantly negatively correlated with perceived stress and anxiety, respectively; and perceived stress was significantly positively correlated with anxiety. The correlations between flow experience and perceived stress, as well as anxiety, did not reach statistical significance.

**TABLE 5:** Descriptive statistics, correlation coefficients, and discriminant validity in group *B* (*N* = 572).

Variable	*M*	*SD*	1	2	3	4
1 Flow experience	14.99	4.36	(0.647)	(0.635)	(0.600)	(0.645)
2 Social connectedness	25.32	7.29	0.251***			
3 Perceived stress	19.28	7.83	−0.068	−0.362***		
4 Anxiety	10.18	4.67	−0.082	−0.195***	0.408***	

Off-diagonal values are Pearson correlation coefficients; values in parentheses on the diagonal are the square roots of AVE. Significance is judged based on *p*-values corrected by the Holm method;

*** *p* < .001.

After correcting the 6 correlation tests for multiple comparisons using the Holm method, the correlations between flow experience and social connectedness, social connectedness and perceived stress, social connectedness and anxiety, and perceived stress and anxiety remained significant, with all corrected *p*-values less than 0.001. The correlation between flow experience and perceived stress was not significant, *r* = −0.068, corrected *p* = 0.102; the correlation between flow experience and anxiety was also not significant, *r* = −0.082, corrected *p* = 0.101. Therefore, H2a, H2d, H2e, and H2f were supported, while H2b and H2c were not supported.

Considering that both practice years and recent practice frequency are ordinal categorical variables, a supplementary Spearman rank correlation analysis was performed. Both practice years and recent practice frequency were significantly positively correlated with flow experience, ρ = 0.263, *p* < 0.001 and ρ = 0.232, *p* < 0.001, respectively. The correlations between these two piano practice indicators and social connectedness, perceived stress, and anxiety did not reach statistical significance, |ρ| ≤ 0.071, *p* ≥ 0.091.

### 4.4 Piano experience group differences

Before the independent sample *t*-test, Levene’s test was used to examine the homogeneity of variance. The Levene’s tests for social connectedness, perceived stress, and anxiety did not reach statistical significance, with *p*-values of 0.741, 0.449, and 0.820, respectively. Therefore, the independent sample *t*-test assuming equal variances was used, and the results are shown in Table 6.

**TABLE 6:** Comparison of psychological variables between group A and group B.

Indicator	Group *A* (*n* = 318) M ± SD	Group *B* (*n* = 572) M ± SD	*t*(888)	*P*	Cohen’s *d*	Mean Diff 95% CI
Social connectedness	23.31 ± 7.27	25.32 ± 7.29	−3.948	< 0.001	−0.276	[−3.012, −1.012]
Perceived stress	21.27 ± 8.08	19.28 ± 7.83	3.603	< 0.001	0.252	[0.909, 3.085]
Anxiety	11.03 ± 4.66	10.18 ± 4.67	2.594	0.010	0.181	[0.206, 1.487]

Group *A* comprises respondents who chose “never learned/completely do not play currently”; Group *B* comprises respondents who chose “have piano experience/currently practicing.” Mean differences are calculated as Group A minus Group *B*. CI is Confidence Interval.

Compared with Group *A*, Group *B* had significantly higher social connectedness scores and significantly lower perceived stress and anxiety scores. Therefore, H1a-H1c were all supported. The absolute values of Cohen’s *d* for the three between-group differences ranged between 0.181 and 0.276, indicating small effect sizes overall.

**FIGURE 3:**
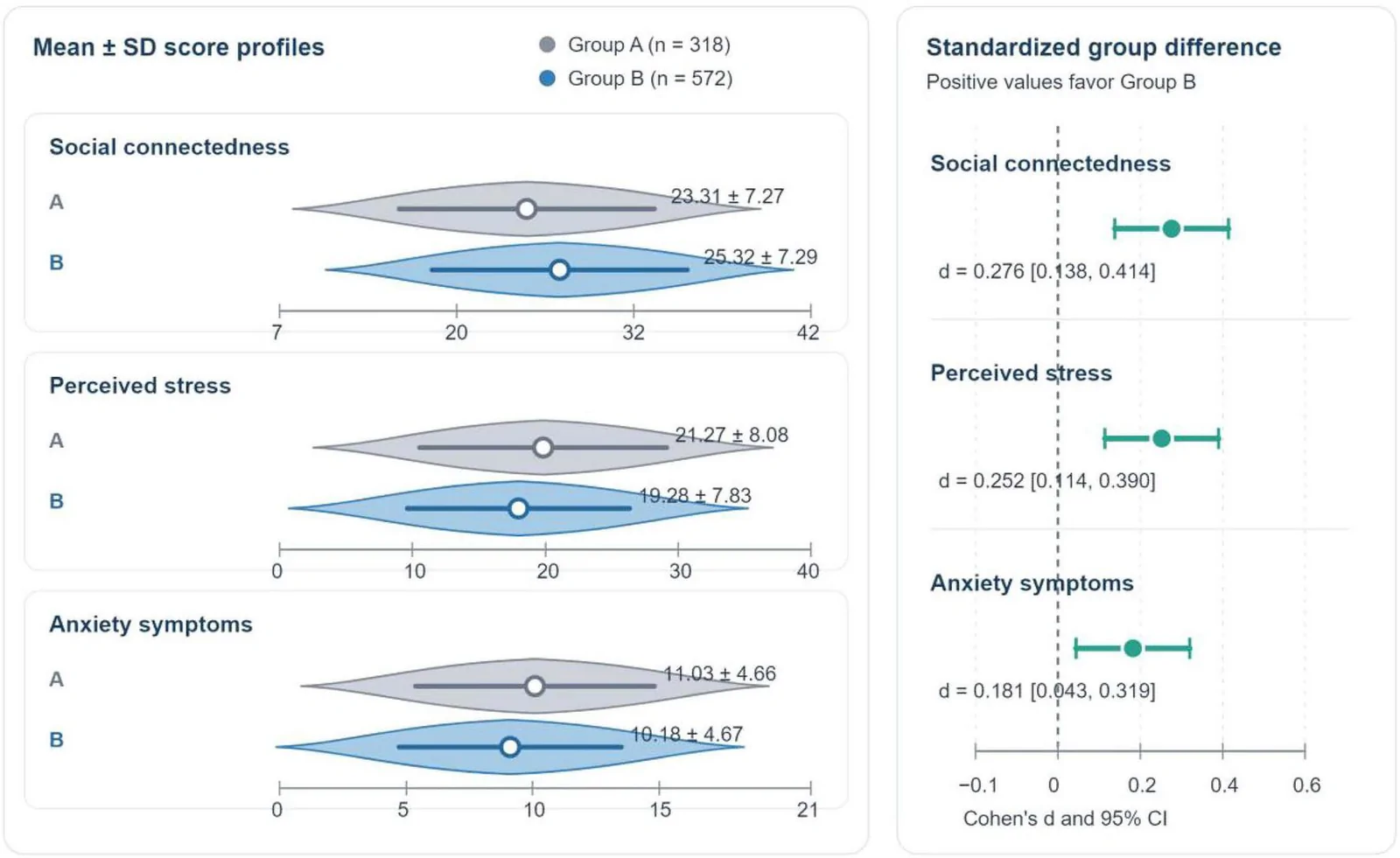
Score distributions and standardized effect sizes of psychological indicators for the two groups. The dots on the left represent within-group means, and horizontal lines represent mean ± standard deviation. The forest plot on the right displays the standardized effect sizes (Cohen’s *d*) of the between-group differences and their 95% confidence intervals. To maintain visual intuition and logical consistency, the direction of effect sizes has been uniformly converted to health-benefit orientation (Positive values favor Group *B*): i.e., positive values on the right side of the vertical axis uniformly indicate that the between-group differences “favor Group B’s performance in relevant mental health dimensions” (manifested as Group *B* having higher social connectedness, or lower perceived stress and anxiety).

The score distributions and standardized effect sizes for the psychological indicators of both groups are shown in Figure 3. Although all between-group differences reached statistical significance, the score distributions of the two groups highly overlapped, and the effect sizes were generally small.

### 4.5 Specific serial indirect association

The serial indirect effect model was conducted in Group *B*, with flow experience as the independent variable, social connectedness as the first mediating variable, perceived stress as the second mediating variable, and anxiety as the outcome variable, controlling for gender, education level, and occupational status. The main path results in each regression equation are shown in Table 7.

**TABLE 7:** Regression paths of the serial model.

Outcome variable	Predictor variable	*B*	*SE*	*t*	*P*	95% CI
Social connectedness	Flow experience	0.426	0.068	6.256	< 0.001	[0.292, 0.559]
Perceived stress	Flow experience	0.041	0.073	0.560	0.576	[−0.102, 0.184]
Perceived 0tress	Social connectedness	−0.396	0.044	−9.078	< 0.001	[−0.482, −0.311]
Anxiety	Flow experience	−0.050	0.042	−1.177	0.240	[−0.133, 0.033]
Anxiety	Social connectedness	−0.024	0.027	−0.890	0.374	[−0.078, 0.029]
Anxiety	Perceived stress	0.232	0.025	9.460	< 0.001	[0.184, 0.281]

The model controls for gender, education level, and occupational status. B is the unstandardized regression coefficient, CI is Confidence Interval.

**FIGURE 4:**
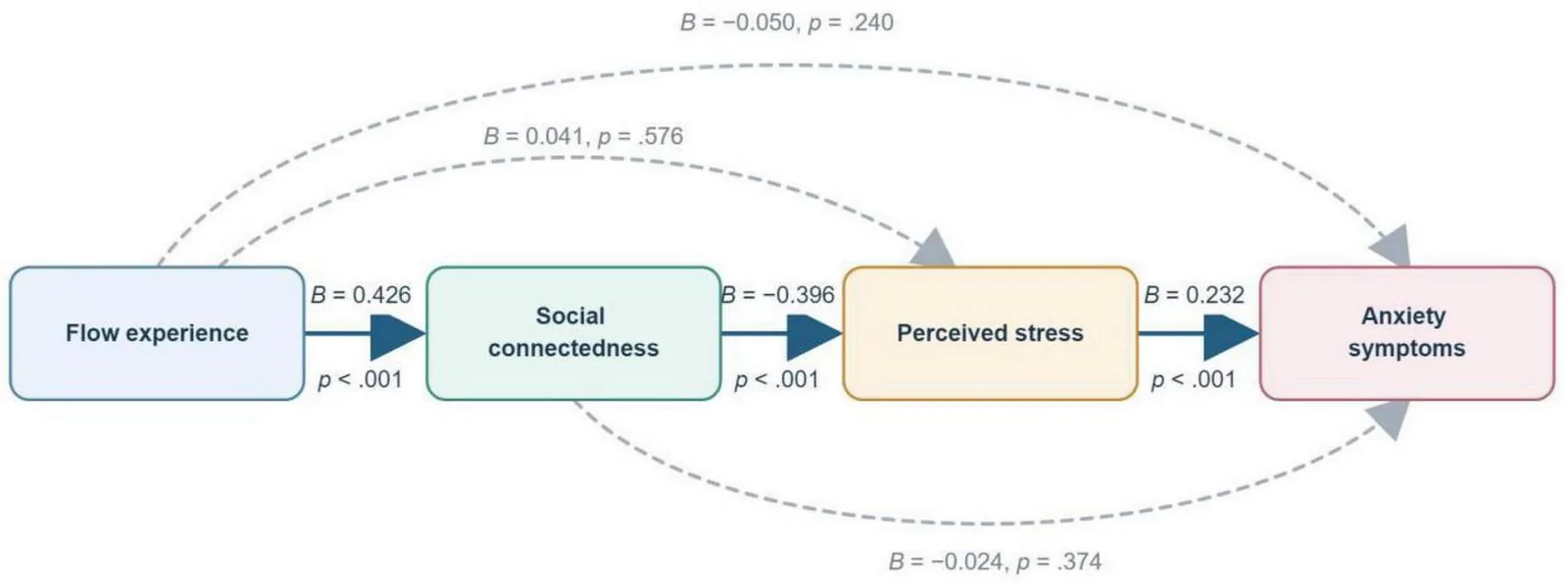
Cross-sectional association paths of core variables in group *B.* The values in the figure are unstandardized regression coefficients B. Solid lines represent paths that reached statistical significance, while dashed lines represent paths that did not reach statistical significance. The model controls for gender, education level, and occupational status. Arrows denote statistical order under theoretical settings, not denoting time sequence or causal relationships.

Flow experience showed a significant positive association with social connectedness, social connectedness showed a significant negative association with perceived stress, and perceived stress showed a significant positive association with anxiety. The conditional direct association between flow experience and perceived stress, as well as the conditional direct association between flow experience, social connectedness, and anxiety, did not reach statistical significance. The *R*^2^ for the three regression equations with social connectedness, perceived stress, and anxiety as outcome variables were 0.075, 0.141, and 0.181, respectively. After controlling for gender, education level, and occupational status, the conditional association paths among the core variables in the serial model are shown in Figure 4.

The Bootstrap test results of various direct and indirect effects are shown in Table 8. Among the three specific indirect paths, only the serial indirect effect of “flow experience → social connectedness → perceived stress → anxiety” reached statistical significance.

**TABLE 8:** Bootstrap effect decomposition of the serial model (5,000 Resamples).

Effect Path	*B*	BootSE	95% Bootstrap CI
Direct effect of flow experience → anxiety	−0.050	0.042	[−0.132, 0.032]
Flow experience → social connectedness → anxiety	−0.010	0.011	[−0.033, 0.011]
Flow experience → perceived stress → anxiety	0.009	0.017	[−0.023, 0.044]
Flow experience → social connectedness → perceived stress → anxiety	−0.039	0.009	[−0.059, −0.023]
Total indirect effect	−0.040	0.021	[−0.082, 0.001]
Total effect	−0.090	0.045	[−0.178, −0.002]

The model controls for gender, education level, and occupational status. CI is the 95% Bootstrap Percentile Confidence Interval; not containing 0 means the corresponding effect reached statistical significance.

The specific serial indirect effect of “flow experience → social connectedness → perceived stress → anxiety” was *B* = −0.039, with a 95% Bootstrap CI of [−0.059, −0.023], and the confidence interval did not contain 0. Therefore, H3 was supported at this specific serial path level. The other two specific indirect effects were insignificant, and the confidence interval for the total indirect effect also contained 0. The direct effect between flow experience and anxiety was not significant, but the total effect reached statistical significance.

Overall, among the three specific indirect paths simultaneously estimated in the model, only the serial indirect association sequentially formed via social connectedness and perceived stress received statistical support. Given that this study utilized a cross-sectional survey design, this result reflects a statistical indirect association between variables and does not represent time sequence or causal relationships.

### 4.6 Moderation test of practice years

The moderation model took social connectedness as the dependent variable and controlled for gender, education level, and occupational status. First, practice years were coded as an ordinal variable from 1 to 4, and an interaction term was constructed after mean centering flow experience and practice years; subsequently, practice years were treated as a categorical variable with “within 1 year” as the reference group to conduct sensitivity analysis. The test results of both models are shown in Table 9.

**TABLE 9:** Moderation test of practice years on the relationship between flow experience and social connectedness (Group *B*, *N* = 572).

Practice years handling method	Test term	*B*	*SE*	*t/F*	*p*	95% CI	Δ*R*^2^
Ordinal variable (1–4)	Flow experience	0.424	0.071	5.971	< 00.001	[0.285, 0.563]	–
	Practice years	−0.050	0.287	−0.176	0.861	[−0.613, 0.513]	–
	Flow experience × practice years	0.059	0.065	0.906	0.365	[−0.069, 0.186]	0.001
Categorical variable	Flow experience × 1–3 years	0.154	0.180	0.855	0.393	[−0.200, 0.507]	–
	Flow experience × 3–5 years	0.239	0.217	1.101	0.271	[−0.187, 0.664]	–
	Flow experience × over 5 years	0.081	0.211	0.385	0.701	[−0.334, 0.496]	–
	Joint test of three interaction terms	–	–	*F*(3,557) = 0.469	0.704	–	0.002

The dependent variable is social connectedness. The models control for gender, education level, and occupational status; the categorical variable model uses “within 1 year” as the reference group. The continuous variable interaction model’s *R*^2^ = 0.076, and the categorical variable interaction model’s *R*^2^ = 0.082. B is the unstandardized regression coefficient, CI is Confidence Interval.

When treating practice years as an ordinal variable, flow experience showed a significant positive association with social connectedness, but the main effect of practice years and the interaction effect between flow experience and practice years were both insignificant, and the explanation rate increment brought by the interaction term was Δ*R*^2^ = 0.001. After using practice years as a categorical variable, the three interaction terms and their joint test also did not reach statistical significance. The joint test result was *F*(3, 557) = 0.469, *p* = 0.704, Δ*R*^2^ = 0.002.

**FIGURE 5:**
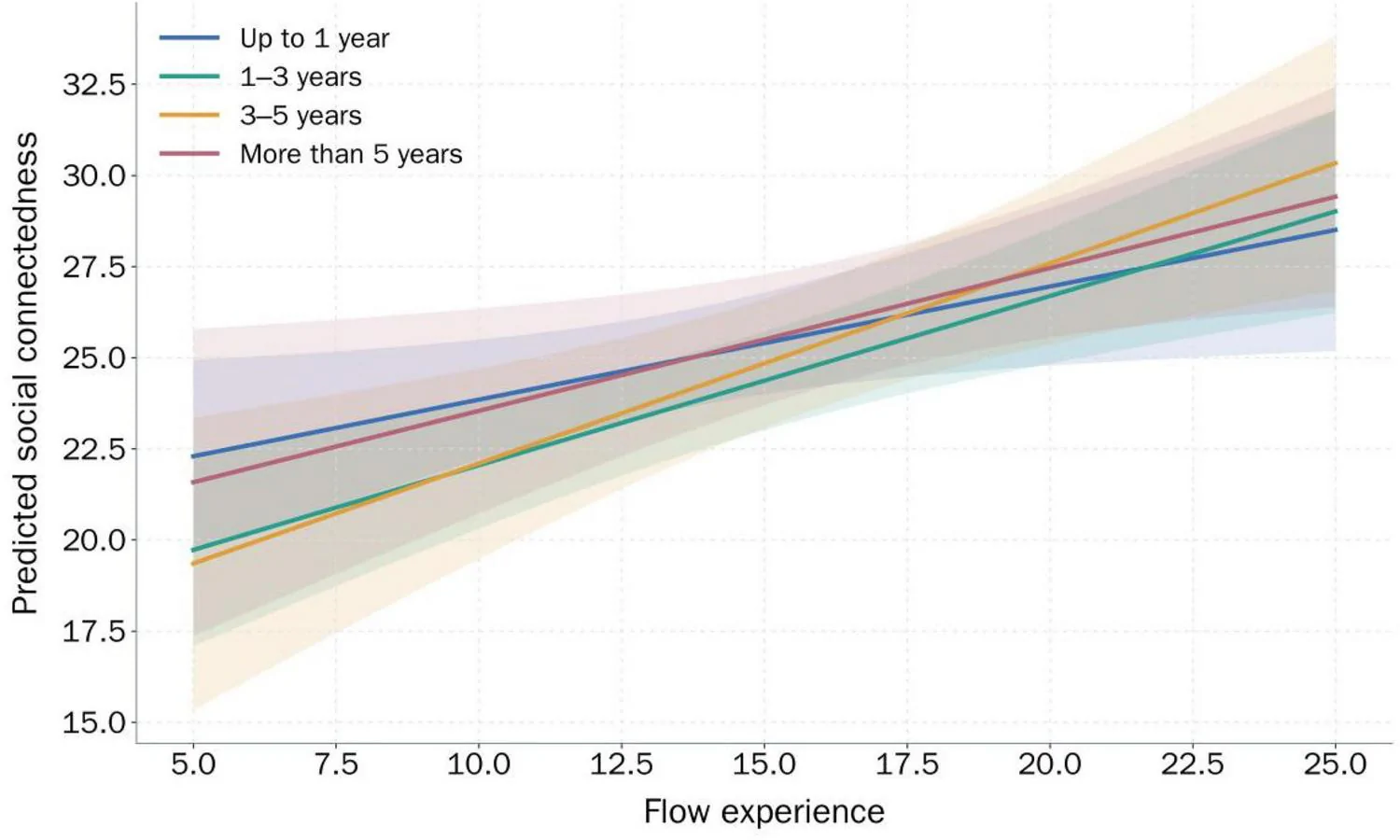
Simple slope prediction plot of the relationship between flow experience and social connectedness across different practice year groups. The vertical axis shows the predicted values of social connectedness after controlling for gender, education level, and occupational status, while the shaded areas represent the 95% confidence bands. The prediction results stem from the categorical variable regression model with “within 1 year” as the reference group. The prediction lines are used to present the conditional association estimated by the model, not denoting causal relationships.

To intuitively present the conditional associations across different practice year groups, the adjusted predicted values and their 95% confidence bands calculated according to the categorical variable model are shown in Figure 5. All four prediction lines showed a positive trend, and their slopes were generally close, with widely overlapping confidence bands, consistent with the result that the interaction terms and their joint tests did not reach statistical significance.

Therefore, H4 was not supported. The current data did not find that practice years significantly moderate the relationship between flow experience and social connectedness.

## 5 Discussion

### 5.1 Main findings

Based on 890 valid questionnaires, this study examined the cross-sectional relationships among piano experience coding groups and social connectedness, perceived stress, and anxiety, and analyzed the associations between flow experience and the aforementioned variables within Group *B*. Group *B*’s social connectedness was higher than Group *A*’s, and perceived stress and anxiety were lower than Group *A*’s, but the effect sizes of the group differences were all small. Within Group *B*, flow experience was positively correlated with social connectedness, but its bivariate correlations with perceived stress and anxiety did not reach significant levels. The serial model only supported the specific indirect association of “flow experience → social connectedness → perceived stress → anxiety,” while the total indirect effect did not reach a significant level; the moderation effect of practice years was also not supported. Overall, the research results were consistent with some expectations in the theoretical model, but the related findings should be restricted to cross-sectional statistical associations. The results provided important empirical clues for understanding the positive associations between daily arts participation and mental health indicators (Fancourt et al., 2020; Mak et al., 2021).

### 5.2 Flow experience and social connectedness

Flow experience was positively correlated with social connectedness, indicating that among Group B respondents, those reporting stronger piano performance flow experience usually also reported higher general social connectedness. Flow experience involves concentration of attention, merging of action and awareness, and the intrinsic experience brought by the activity itself (Jackson et al., 2008), whereas social connectedness reflects the individual’s feelings of belonging, understanding, and acceptance in interpersonal relationships (Lee and Robbins, 1995). Although typical performance flow is usually viewed as a focused state in a specific context, while social connectedness reflects relatively stable psychological appraisals in daily life, the correlation between the two illustrates that even subjective engagement states in specific activities may have statistical covariation with an individual’s overall feelings about social relationships (Savage et al., 2020).

Piano activities can be individual practices or involve teaching, collaborative performance, public performance, and experience exchange. Teacher-student interactions, peer feedback, and family support in different contexts may be simultaneously correlated with flow experience and social connectedness (Perkins et al., 2021). Those with higher social connectedness may also possess more participation opportunities and support resources, thereby reporting better performance experiences in the questionnaire. Additionally, unmeasured variables such as positive emotion, openness, engagement tendency, and self-efficacy may also be correlated with these two measurement outcomes (Kim et al., 2023).

This study did not measure specific performance forms, repertoire types (e.g., Western classical versus traditional Chinese pieces), interaction frequency, social support, and music identity; therefore, it cannot be determined which activity contexts this correlation primarily corresponds to. Future research could distinguish between individual practice, instruction, collaborative performance, and public performance, and include indicators such as interaction frequency, social support, and belonging experience, to further describe the relationship between flow experience and social connectedness.

### 5.3 Social connectedness, perceived stress, and anxiety

Social connectedness was negatively correlated with perceived stress, and had a weaker negative correlation with anxiety; perceived stress was positively correlated with anxiety. Respondents with stronger feelings of belonging, understanding, and interpersonal connection usually reported lower stress and anxiety, whereas those with higher stress perception often reported more distinct anxiety.

The link between social connectedness and perceived stress may involve social support, stress appraisal, and social participation. According to the stress-buffering hypothesis of social support (Cohen and Wills, 1985), the social network characteristics of individuals with higher social connectedness may be associated with more positive subjective appraisals when facing life loads (Perry et al., 2024); conversely, those with higher stress and anxiety may also exhibit less social participation or more negative interpersonal appraisals in daily life. Current cross-sectional data cannot distinguish the direction of these relationships.

Furthermore, while the bivariate correlation between social connectedness and anxiety reached significant levels, the conditional direct association between the two was no longer significant after incorporating perceived stress. This indicates that the correlation between social connectedness and anxiety may partly correspond to their covariation with perceived stress, but it cannot be asserted that the relationship between the two is entirely explained by perceived stress.

The degree of correlation between perceived stress and anxiety was higher than that between social connectedness and anxiety, demonstrating that the appraisal of unpredictability, uncontrollability, and psychological load of life events (Cohen et al., 1983) has a tighter cross-sectional connection with recent anxiety. The two measurements may also have some overlap in psychological tension and negative experiences, and this measurement characteristic needs to be considered when interpreting the correlation results.

### 5.4 Specific serial indirect association

In the serial model, the confidence interval of the specific indirect effect of “flow experience → social connectedness → perceived stress → anxiety” did not contain 0. According to the model settings, stronger flow experience is correlated with higher social connectedness (Habe et al., 2021), higher social connectedness is correlated with lower perceived stress, and lower perceived stress is further correlated with less anxiety (Thoma et al., 2013). This result provided a potential theoretical clue for understanding the joint relationship among the four variables, suggesting that the association between arts participation on emotional states is not a simple direct point-to-point mapping. Instead, it operates through a complex cascade network where cumulative social resources (social connectedness) and cognitive remodeling (perceived stress reduction) serve as interlocked transmission gears (Dingle et al., 2021).

This result needs to be judged in combination with other effects. The two specific indirect effects formed by flow experience via social connectedness or perceived stress respectively were insignificant, and the confidence interval of the total indirect effect also contained 0. Therefore, statistical support was only concentrated on the specific serial path containing both social connectedness and perceived stress simultaneously, which cannot be expanded to establish the overall indirect effect.

The bivariate correlation and conditional direct association between flow experience and anxiety were both insignificant. Although the total effect after controlling for covariates reached a significant level, its upper confidence interval limit was close to 0, and was not completely consistent with the bivariate correlation results, indicating that the net association between the two was weak and might fluctuate with model settings and covariate adjustments. The arrows in the serial model merely represent statistical ordering and variance decomposition based on theoretical hypotheses, by no means designating confirmed time sequence or causal directions (Hayes, 2022). Flow experience, social connectedness, perceived stress, and anxiety were all measured at the same time point; other arrangements and unmeasured variables could still act as confounding factors to explain the observed statistical relationships. Subsequent research needs to adopt multi-timepoint tracking or intensive measurement to investigate the actual co-variation trajectories of these variables across different times and performance contexts.

### 5.5 Practice years and its moderating effect

Practice years were positively correlated with flow experience, showing that respondents with longer practice experience usually reported stronger typical performance flow experience. The accumulation of experience may have a concomitant association with performance proficiency, repertoire selection preferences, and challenge-skill balance (Csikszentmihalyi, 1990), but this study did not directly measure these indicators, and related explanations still require further empirical corroboration.

Practice years were not significantly correlated with social connectedness, perceived stress, and anxiety. Whether treating practice years as an ordinal or categorical variable, its interaction term with flow experience also did not reach significance. The current data did not show that the relationship between flow experience and social connectedness stably strengthened or weakened with practice years.

Practice years can only reflect duration of experience, not fully representing current participation levels, cumulative investment, and performance proficiency. Respondents with the same practice years might differ in learning continuity, practice frequency, course experience, skill level, and performance forms. The open-ended category of “over 5 years” also enlarged within-group heterogeneity. Future research could record exact years of learning, interruption experiences, cumulative practice hours, recent practice investment, skill levels, and repertoire difficulty to improve the precision of piano experience measurement.

### 5.6 Boundary of interpretation for piano experience group differences

Group *B*’s social connectedness was higher than Group *A*’s, and perceived stress and anxiety were lower than Group *A*’s, but the effect sizes for the group differences on all three indicators were small. Although contemporary methodological research points out that in complex psychological phenomena determined by multiple factors, small effect sizes are equally real and possess cumulative public health significance (Götz et al., 2022), this still means that the score distributions on various mental health scales for the two groups have massive overlapping intervals. Given the large sample size (*N* = 890), it is vital to distinguish between a statistically detectable group difference and evidence of a substantial, clinically meaningful mental-health benefit caused by piano participation itself. Therefore, it is inappropriate to arbitrarily predict or categorize an individual’s overall psychological condition directly based on the questionnaire coding group of specific instruments (Newman et al., 2022).

Respondents’ piano learning experiences may simultaneously correlate with personal interests, family resources, educational background, disposable time, social support, and preexisting psychological states. Those with more social support or ample time conditions may have more opportunities to participate in piano activities, whereas those with higher stress might reduce or stop practicing. The current survey did not collect psychological indicators prior to the onset of piano activities, nor could it adequately handle such self-selection factors.

The grouping options also restricted the meaning of between-group comparisons. Group *A*’s “never learned/completely do not play currently” simultaneously contained never-learners and those who currently stopped playing, while Group *B*’s “have piano experience/currently practicing” might contain those with only past experience and those currently practicing. The two groups were not a strict division between “those with no piano experience” and “those currently practicing regularly,” and related results should be restricted to differences between questionnaire coding groups.

Subsequent surveys could set “never learned,” “learned in the past but currently stopped,” “currently practice occasionally,” and “currently practice regularly” as mutually exclusive categories, and record the time and reasons for stopping practice to enhance the clarity of group definitions.

### 5.7 Research limitations and future directions

This study adopted a cross-sectional design, where primary variables were measured at the same time point, making it impossible to determine the time sequence between variables (Hayes, 2022). Group differences, correlations, and serial indirect effects should all be interpreted as statistical associations. Subsequent research could adopt longitudinal tracking or intensive measurement to record the covariation of piano activity participation status and psychological indicators at different time points.

The research data mainly came from self-reports. Respondents’ answers regarding typical flow states, stress over the past month (Cohen et al., 1983), and anxiety over the past 2 weeks (Spitzer et al., 2006) might be interfered with by recall bias, current emotions, and social desirability tendencies. Although Harman’s single-factor test did not indicate a dominant method factor, this procedure provides only a preliminary assessment. Because all core variables were measured synchronously from the same source, common-method bias cannot be entirely ruled out and should be treated as an unresolved design limitation. Future studies could combine practice logs, behavioral tasks, teacher evaluations, and physiological indicators (such as cortisol and other objective stress markers; Perry et al., 2024) to increase measurement sources.

The measurement precision for piano experience groups and practice years was limited and failed to fully reflect current participation status, learning continuity, cumulative investment, and actual performance level. Subsequent questionnaires should separately record learning experience, current practice status, practice frequency, duration of a single practice, cumulative practice volume, skill levels, and primary performance forms.

The statistical models controlled for gender, education level, and occupational status, but several theoretically relevant confounding variables, such as age, socioeconomic resources, region, life events, pre-existing social support, personality traits, other arts activities, and motivation for participating in piano activities, were not included. This limitation is particularly consequential for the between-group comparisons, as access to piano education may inherently correlate with socioeconomic, familial, and cultural resources that independently foster social connectedness and psychological wellbeing. Consequently, the observed group differences cannot presently be attributed specifically to piano experience. Subsequent research could perfect variable measurement and covariate selection based on pre-set theoretical frameworks.

Some measurement tools were simplified or adjusted to fit the research context. The internal consistency and composite reliability of each scale reached acceptable levels, and the overall fit of the four-factor model met common standards, but the AVE for each construct was below 0.50, meaning convergent validity remained limited (Fornell and Larcker, 1981). Individual items in the brief Social Connectedness Scale also have room for expression improvement. Subsequent studies could adjust items through cognitive interviews and larger-scale psychometric validation, and item analysis, and test the scale structure in independent samples.

This study collected data through an online questionnaire platform. The sample composition might be affected by distribution channels, voluntary participation, and internet usage habits. The application scope of the research results should be limited to populations akin to the current sample characteristics. Follow-up studies could examine the stability of results through multi-channel recruitment and replication in different samples.

## 6 Conclusion

This study examined the cross-sectional relationships among piano experience coding groups, piano performance flow experience, social connectedness, perceived stress, and anxiety. Results indicated that individuals with current or past piano participation profiles (Group *B*) exhibited statistically significant, albeit small, advantages in mental health indicators compared to those without such regular participation baseline (Group *A*): Group *B*’s social connectedness was higher, and their perceived stress and anxiety were lower. Constrained by research design, grouping methods, and self-selection factors, these results merely reflected statistical differences between questionnaire coding groups and cannot be used to deduce the overall psychological homogeneity of groups with specific instrument experience (Newman et al., 2022).

Within Group *B*, flow experience was positively correlated with social connectedness, but its bivariate correlations with perceived stress and anxiety did not reach significant levels. The specific serial indirect effect of “flow experience → social connectedness → perceived stress → anxiety” obtained statistical support, but other specific indirect effects and total indirect effects did not reach significant levels. Practice years were positively correlated with flow experience, but did not significantly moderate the relationship between flow experience and social connectedness.

Overall, piano performance flow experience did not show simple cross-sectional direct bivariate associations with psychological indicators, but possessed partial multi-node network covariation relationships with social connectedness, perceived stress, and anxiety that fit theoretical expectations (Dingle et al., 2021). While the relevant results provide clues for subsequent explorations of daily intervention mechanisms, they inherently represent statistical covariations rather than established causal mechanisms, and thus cannot confirm the time sequence between variables. Future research needs to adopt clearer piano experience groupings, more refined practice indicators, as well as multi-timepoint and multi-source data to further test the dynamic evolution rules of these associations (Agres and Chen, 2025; Mak et al., 2021).

## Data Availability

The original contributions presented in the study are included in the article/Supplementary material, further inquiries can be directed to the corresponding author.
