# Supplementary material for: The association pathway between music practice and mental health: a study on social connectedness, perceived stress, and anxiety among individuals with piano experience
Source: Front Psychol. 2026 Sep 8;17:1957899. doi: 10.3389/fpsyg.2026.1957899 (PMC13593876; doi:10.3389/fpsyg.2026.1957899)
Supplement: Supplementary file 1 [file Data_Sheet_1.zip › Supplementary_Material_Survey_Codebook.docx]

# Supplementary Material (补充材料)

**Title:** Survey Instrument for Exploring Psychological Healing Pathways Through Music Interventions

**文档名称：** 探寻音乐干预心理疗愈途径的调查问卷工具

**Note to Reviewers (致审稿人的说明):**

1. The survey was originally administered in Chinese constraint context. The English translation is provided here for review purposes. (本问卷在实际调查中采用中文发放，此处提供英文对照以便审阅。)
2. The “(Reverse-scored)” tags indicating reverse scoring were not visible to participants during actual data collection. (题目中提示反向计分的“(反向)”字样在实际数据收集时对受访者不可见。)

### Part 1: Informed Consent (第一部分：知情同意书)

**尊敬的参与者 / Dear Participant:**

您好！我们是进行心理学与公共健康研究的学术科研团队，目前正在开展一项关于“音乐实践（如钢琴练习）对成人日常心理状态、压力应对及社会互动关系”的科学调查。

*Hello! We are an academic research team conducting a study on psychology and public health. We are currently conducting a scientific survey on “the relationship between music practice (e.g., piano practice) and adults’ daily psychological states, stress coping, and social interactions.”*

**隐私与保密说明 (Privacy and Confidentiality):** 本研究采用完全匿名的方式收集数据，您的任何个人可识别信息均不会被记录或关联。数据仅用于总体统计分析和学术论证。

*This study collects data completely anonymously. None of your personally identifiable information will be recorded or linked. Data will only be used for aggregate statistical analysis and academic demonstration.*

**自愿参与 (Voluntary Participation):** 您的参与完全自愿，在答题过程中的任何时刻，您都有权无条件直接关闭页面退出测试。

*Your participation is completely voluntary. At any point during the survey, you have the right to unconditionally close the page and withdraw from the test.*

**参与条件 (Eligibility):** 根据科研伦理准则，本问卷仅面向 年满18周岁 的成年受访者。完成全部问卷约需 5-8 分钟。

*According to research ethics guidelines, this survey is exclusively for adult respondents aged 18 and above. The entire questionnaire takes about 5-8 minutes to complete.*

**1. 您是否已仔细阅读上述知情同意内容，并确认自己已年满18周岁，自愿同意参与本研究？(逻辑跳转题) / Have you carefully read the informed consent above, confirmed you are at least 18 years old, and voluntarily agree to participate in this study? (Skip Logic Item)**

A. 是的，我已年满18周岁并自愿同意（进入问卷正文）/ *Yes, I am 18 or older and voluntarily agree (Proceed to survey)*

B. 否，我不满18周岁或不同意（结束调查）/ *No, I am under 18 or do not agree (Terminate survey)*

### Part 2: Sociodemographic Information (第二部分：基础人口学信息)

**2. 您的生理性别是 (Your biological sex is):**

男 / *Male*

女 / *Female*

**3. 您目前的最高受教育程度及状态 (Your highest level of education and current status):**

高中/中专及以下 / *High school/Vocational school or below*

大专 / *Associate / College diploma*

本科（含在读）/ *Bachelor’s degree (including current students)*

硕士研究生及以上（含在读）/ *Master’s degree or above (including current students)*

**4. 您目前的职业状态 (Your current employment status):**

全职学生 / *Full-time student*

全职工作 / *Employed full-time*

自由职业/兼职 / *Freelance / Part-time*

待业/退休 / *Unemployed / Retired*

### **Part 3: Music Practice Experience and Flow Mechanism (第三部分：练琴行为体验与多感官沉浸机制)**

**5. 您目前是否有过钢琴学习或日常练习的经历？ (系统跳转题) / Do you currently have any experience learning or practicing the piano? (System Skip Logic)**

A. 从未学过/目前完全不弹，直接跳转至第四部分 / *Never learned/Do not play at all, skip to Part 4*

B. 有过经验/目前正在练习，继续答题 / *Have experience/Currently practicing, continue*

*(注：以下 6-12 题仅对第5题选择 B 的受访者显示 / Note: Questions 6-12 below are only displayed to respondents who selected ‘B’ for Q5.)*

**6. 您的钢琴练习总时长（经验累积）大概是多久？/ Roughly, what is your total accumulated time of piano practice?**

1年以内（初学者）/ *Under 1 year (Beginner)*

1-3年 / *1-3 years*

3-5年 / *3-5 years*

5年以上（资深练习者）/ *Over 5 years (Experienced practitioner)*

**7. 在近一个月内，您平均每周进行多少次钢琴弹奏？/ In the past month, how many times per week on average did you practice the piano?**

小于1次 / 偶尔弹一次 / *Less than once / Seldom*

1-2次 / *1-2 times*

3-4次 / *3-4 times*

5次及以上 / *5 times or more*

**【多感官心流体验 / Multisensory Flow Experience】**

**指导语：** 请回想您在全身心弹奏钢琴时的典型状态，根据您的实际感受对以下描述进行评分 (1=完全不同意, 5=完全同意)。

*Instructions: Please recall your typical state when fully immersed in playing the piano, and rate the following statements based on your actual feelings (1=Strongly disagree, 5=Strongly agree).*

| Statements (题目描述) | 1 | 2 | 3 | 4 | 5 |
| --- | --- | --- | --- | --- | --- |
| 8. 弹奏时，我感觉自身能力能够应对所弹奏曲目的挑战。 *When playing, I feel that my abilities correlate with the challenge of the piece.* | ○ | ○ | ○ | ○ | ○ |
| 9. 弹奏不仅要看谱听音，我能感到全身感官（动作与意识）融为了一体。 *Playing is beyond seeing and hearing; I feel my senses, actions, and awareness merge into one.* | ○ | ○ | ○ | ○ | ○ |
| 10. 弹琴时我会十分专注，毫不费力就能保持极高的注意力。 *I am extremely focused and can maintain high attention effortlessly when playing.* | ○ | ○ | ○ | ○ | ○ |
| 11. 弹奏时发生的一切仿佛是自然流露的，我甚至感觉不到时间的流逝。 *Everything seems to happen naturally, and I even lose track of time.* | ○ | ○ | ○ | ○ | ○ |
| 12. 这项体验（弹琴）本身带给我巨大的奖励感和纯粹的愉悦。 *The experience itself brings me a huge sense of reward and pure pleasure.* | ○ | ○ | ○ | ○ | ○ |

### Part 4: Brief Social Connectedness Scale (第四部分：简版社会连接量表 - Brief SCS)

**指导语：** 每个人在这个社会中对人际交往的感觉都不同。请根据您 近期的真实感受，评价以下陈述与您的相符程度。(1=强烈不同意, 6=强烈同意)

*Instructions: Everyone feels differently about interpersonal interactions in society. Based on your recent true feelings, please rate how much you agree with the following statements. (1=Strongly disagree, 6=Strongly agree)*

| Statements (题项描述) | 1 | 2 | 3 | 4 | 5 | 6 |
| --- | --- | --- | --- | --- | --- | --- |
| 13. 即使在人群中，我也经常感到形单影只。(反向 / Reverse-scored) *Even among people, I often feel alone.* | ○ | ○ | ○ | ○ | ○ | ○ |
| 14. 我能与身边的人产生一种亲近、彼此相连的感受。 *I am able to connect closely with the people around me.* | ○ | ○ | ○ | ○ | ○ | ○ |
| 15. 我觉得自己像个局外人，不属于任何一个群体。(反向 / Reverse-scored) *I feel like an outsider, not belonging to any group.* | ○ | ○ | ○ | ○ | ○ | ○ |
| 16. 参与社会互动时，我通常感到安全和自在。 *I usually feel safe and comfortable when participating in social interactions.* | ○ | ○ | ○ | ○ | ○ | ○ |
| 17. 我经常觉得自己跟周围的世界或者是社会的节奏脱节。(反向 / Reverse-scored) *I often feel disconnected from the world around me or the pace of society.* | ○ | ○ | ○ | ○ | ○ | ○ |
| 18. 我认为自己容易被他人接纳和理解。 *I think I am easily accepted and understood by others.* | ○ | ○ | ○ | ○ | ○ | ○ |
| 19. 我感觉到在这个世界上没有人能够真正依靠我。(反向 / Reverse-scored) *I feel that there is no one in this world who I can truly rely on.* | ○ | ○ | ○ | ○ | ○ | ○ |

### Part 5: Perceived Stress Scale (第五部分：压力感知量表 - PSS-10)

**指导语：** 请回忆在 过去的一个月里，您的感受和想法。判断以下情况发生的频率。(0=从不, 1=几乎没, 2=有时, 3=经常, 4=总是)

*Instructions: Please recall your feelings and thoughts during the last month. Indicate how often you felt or thought a certain way. (0=Never, 1=Almost never, 2=Sometimes, 3=Fairly often, 4=Very often)*

| In the last month, how often have you… (在过去一个月里，有几次…) | 0 | 1 | 2 | 3 | 4 |
| --- | --- | --- | --- | --- | --- |
| 20. 因为发生出乎意料的事情而感到心烦意乱？ *…been upset because of something that happened unexpectedly?* | ○ | ○ | ○ | ○ | ○ |
| 21. 感觉自己无法控制生活中重要的事情？ *…felt that you were unable to control the important things in your life?* | ○ | ○ | ○ | ○ | ○ |
| 22. 感到神经紧张和承受重重压力？ *…felt nervous and ‘stressed’?* | ○ | ○ | ○ | ○ | ○ |
| 23. 能够成功应对生活中令人烦恼的烂摊子？(反向 / Reverse) *…handled irritating life hassles successfully?* | ○ | ○ | ○ | ○ | ○ |
| 24. 觉得正在发生的一切都在自己掌控之中？(反向 / Reverse) *…felt that things were going your way?* | ○ | ○ | ○ | ○ | ○ |
| 25. 发现自己无法应付那些必须要完成的事情？ *…found that you could not cope with all the things that you had to do?* | ○ | ○ | ○ | ○ | ○ |
| 26. 能够控制住生活中的烦躁情绪？(反向 / Reverse) *…been able to control irritations in your life?* | ○ | ○ | ○ | ○ | ○ |
| 27. 觉得一切尽在掌握？(反向 / Reverse) *…felt that you were on top of things?* | ○ | ○ | ○ | ○ | ○ |
| 28. 碰到事情时会因为超出自己控制而有些发火？ *…been angered because of things that were outside of your control?* | ○ | ○ | ○ | ○ | ○ |
| 29. 觉得困难的事情越积越多，多到无法摆脱？ *…felt difficulties were piling up so high that you could not overcome them?* | ○ | ○ | ○ | ○ | ○ |

### Part 6: Generalized Anxiety Disorder Scale (第六部分：日常焦虑水平检测 - GAD-7)

**指导语：** 在 过去的 2 周内，您有多少天受到以下各个问题的困扰？(0=一天也没有, 1=少数几天, 2=一半以上天数, 3=几乎每天)

*Instructions: Over the last 2 weeks, how often have you been bothered by the following problems? (0=Not at all, 1=Several days, 2=More than half the days, 3=Nearly every day)*

| Problems (困扰问题描述) | 0 | 1 | 2 | 3 |
| --- | --- | --- | --- | --- |
| 30. 感觉难以平静、内心焦虑或急躁 *Feeling nervous, anxious or on edge* | ○ | ○ | ○ | ○ |
| 31. 无法停止或控制对事情的各种担忧 *Not being able to stop or control worrying* | ○ | ○ | ○ | ○ |
| 32. 对各种各样的事情担忧过多 *Worrying too much about different things* | ○ | ○ | ○ | ○ |
| 33. 发现自己很难真正的放松下来 *Trouble relaxing* | ○ | ○ | ○ | ○ |
| 34. 烦躁得坐立不安，难以保持安静 *Being so restless that it is hard to sit still* | ○ | ○ | ○ | ○ |
| 35. 变得非常容易被惹恼，或极度易怒 *Becoming easily annoyed or irritable* | ○ | ○ | ○ | ○ |
| 36. 感到极为害怕，似乎将有可怕的事情发生 *Feeling afraid as if something awful might happen* | ○ | ○ | ○ | ○ |

### Supplementary Material: Data Codebook

#### 补充材料：问卷数据编码本

| Variable Name (变量名) | Item Description (题目描述) | Value Options (赋值选项) | Measure Type (测量类型) | Scoring / Skip Logic (计分与逻辑说明) |
| --- | --- | --- | --- | --- |
| Part 1: Sociodemographic Information (第一部分：基础人口学信息) |  |  |  |  |
| Consent | Confirmed as an adult participant aged 18+ (是否确认为年满18周岁的自愿参与者) | 1 = Yes (是) 2 = No (否) | Nominal (名义变量) | If 2 is selected, survey terminates and is excluded as invalid data. (若选择2，问卷终止并作为无效数据剔除。) |
| Gender | Biological Sex (生理性别) | 1 = Male (男) 2 = Female (女) | Nominal (名义变量) | None (无) |
| Edu_Level | Highest education level completed (最高受教育程度) | 1 = High school or below (高中/中专及以下) 2 = Associate degree (大专) 3 = Bachelor’s degree (本科) 4 = Master’s degree or above (硕士及以上) | Ordinal (定序变量) | None (无) |
| Job_Status | Current employment status (目前职业状态) | 1 = Full-time student (全职学生) 2 = Full-time employee (全职工作) 3 = Freelance / Part-time (自由职业/兼职) 4 = Unemployed / Retired (待业/退休) | Nominal (名义变量) | None (无) |
| Part 2: Piano Practice Experience and Flow (第二部分：钢琴练习经历与心流体验) |  |  |  |  |
| Piano_Exp | Experience with piano learning or daily practice (是否有过钢琴学习或日常练习经历) | 1 = Never learned / Do not play currently (从未学过/目前完全不弹) 2 = Have experience / Currently practicing (有过经验/目前正在练习) | Nominal (名义变量) | If 1 is selected, Piano_Years, Piano_Freq, and Flow_ items are recorded as System Missing. Skip to Brief SCS. (若选1，则后续钢琴年限、频率及心流题记录为缺失值，跳转至社会连接感知量表。) |
| Piano_Years | Total duration of piano practice in years (钢琴练习总时长/年限) | 1 = < 1 year (1年以内) 2 = 1–3 years (1-3年) 3 = 3–5 years (3-5年) 4 = > 5 years (5年以上) | Ordinal (定序变量) | Only valid if Piano_Exp = 2. (仅当Piano_Exp=2时有效。) |
| Piano_Freq | Weekly practice frequency in the recent month (近一个月每周练习频率) | 1 = < 1 time / Occasionally (小于1次/偶尔) 2 = 1–2 times (1-2次) 3 = 3–4 times (3-4次) 4 = ≥ 5 times (5次及以上) | Ordinal (定序变量) | Only valid if Piano_Exp = 2. (仅当Piano_Exp=2时有效。) |
| Flow_1 to Flow_5 | Piano Performance Flow Experience (5 items) (钢琴演奏心流体验，共5题) | 1 = Strongly disagree (完全不同意) … 5 = Strongly agree (完全同意) | Scale (连续变量) | Total Flow Score = Sum(Flow_1 to Flow_5) (心流总分 = 5个题项分值之和) |
| Part 3: Brief Social Connectedness Scale (Brief SCS) (第三部分：简版社会连接感知量表) Scale (标度): 1 = Strongly disagree (强烈不同意) to 6 = Strongly agree (强烈同意) |  |  |  |  |
| SCS_1_Rev | Often feel lonely/isolated (经常感到形单影只) | 1 to 6 | Scale (连续变量) | Reverse scoring (Converted value = $7-\text{original}$) (反向计分：转换值 = 7 - 原始值) |
| SCS_2 | Feel a sense of closeness and mutual connection (产生一种亲近、彼此相连的感受) | 1 to 6 | Scale (连续变量) | Forward scoring (正向计分) |
| SCS_3_Rev | Feel like an outsider (觉得自己像个局外人) | 1 to 6 | Scale (连续变量) | Reverse scoring (Converted value = $7-\text{original}$) (反向计分：转换值 = 7 - 原始值) |
| SCS_4 | Feel safe and comfortable during social interactions (参与社会互动时感到安全和自在) | 1 to 6 | Scale (连续变量) | Forward scoring (正向计分) |
| SCS_5_Rev | Feel out of touch with the rhythm of society (觉得跟社会的节奏脱节) | 1 to 6 | Scale (连续变量) | Reverse scoring (Converted value = $7-\text{original}$) (反向计分：转换值 = 7 - 原始值) |
| SCS_6 | Easily accepted and understood by others (容易被他人接纳和理解) | 1 to 6 | Scale (连续变量) | Forward scoring (正向计分) |
| SCS_7_Rev | No one to truly rely on me (没有人能够真正依靠我) | 1 to 6 | Scale (连续变量) | Reverse scoring (Converted value = $7-\text{original}$) (反向计分：转换值 = 7 - 原始值) |
| SCS_Total | Total score for Social Connectedness (社会连接感总分) | 7 to 42 | Scale (连续变量) | SCS_Total = Sum of all SCS items (including reversed) (所有SCS题项包括反转后的分值之和) |
| Part 4: Perceived Stress Scale (PSS-10) (第四部分：压力感知量表) Scale (标度): 0 = Never (从不) to 4 = Very often (总是) |  |  |  |  |
| PSS_1 | Upset because of unexpected events (出乎意料的事情感到心烦意乱) | 0 to 4 | Scale (连续变量) | Forward scoring (正向计分) |
| PSS_2 | Unable to control important things in life (无法控制生活中重要的事情) | 0 to 4 | Scale (连续变量) | Forward scoring (正向计分) |
| PSS_3 | Felt nervous and “stressed” (神经紧张和承受重重压力) | 0 to 4 | Scale (连续变量) | Forward scoring (正向计分) |
| PSS_4_Rev | Successfully dealt with irritating life hassles (成功应对令人烦恼的烂摊子) | 0 to 4 | Scale (连续变量) | Reverse scoring (Converted value = $4-\text{original}$) (反向计分：转换值 = 4 - 原始值) |
| PSS_5_Rev | Felt things were going your way (一切都在自己掌控之中) | 0 to 4 | Scale (连续变量) | Reverse scoring (Converted value = $4-\text{original}$) (反向计分：转换值 = 4 - 原始值) |
| PSS_6 | Found that you could not cope with all the things (无法应付必须要完成的事情) | 0 to 4 | Scale (连续变量) | Forward scoring (正向计分) |
| PSS_7_Rev | Been able to control irritations in your life (控制住生活中的烦躁情绪) | 0 to 4 | Scale (连续变量) | Reverse scoring (Converted value = $4-\text{original}$) (反向计分：转换值 = 4 - 原始值) |
| PSS_8_Rev | Felt that you were on top of things (觉得一切尽在掌握) | 0 to 4 | Scale (连续变量) | Reverse scoring (Converted value = $4-\text{original}$) (反向计分：转换值 = 4 - 原始值) |
| PSS_9 | Been angered because of things outside your control (超出控制而有些发火) | 0 to 4 | Scale (连续变量) | Forward scoring (正向计分) |
| PSS_10 | Difficulties were piling up so high (困难越积越多无法摆脱) | 0 to 4 | Scale (连续变量) | Forward scoring (正向计分) |
| PSS_Total | Total score for Perceived Stress (压力感知总分) | 0 to 40 | Scale (连续变量) | PSS_Total = Sum of all PSS items (including reversed) (所有PSS题项包括反转后的分值之和) |
| Part 5: Generalized Anxiety Disorder Scale (GAD-7) (第五部分：日常焦虑量表) Scale (标度): 0 = Not at all (没有) to 3 = Nearly every day (几乎每天) |  |  |  |  |
| GAD_1 to GAD_7 | Includes 7 symptoms (e.g., trouble relaxing, not being able to stop worrying) (包含难以平静、停止担忧等7项症状表现) | 0 to 3 | Scale (连续变量) | Forward scoring (正向计分) |
| GAD_Total | Total score for Generalized Anxiety (日常焦虑总分) | 0 to 21 | Scale (连续变量) | GAD_Total = Sum(GAD_1 to GAD_7) (7个日常焦虑题项分值之和) |
